# Three Cases of Intra-articular Metastasis of the Shoulder From Lung Cancer Successfully Treated With Palliative Stereotactic Body Radiotherapy Using CyberKnife

**DOI:** 10.7759/cureus.92303

**Published:** 2025-09-14

**Authors:** Shinichiro Mizumatsu, Hiroshi Ryu

**Affiliations:** 1 Department of Radiation Oncology, Narita Memorial Hospital, Toyohashi, JPN; 2 Department of Neurological Surgery, Narita Memorial Hospital, Toyohashi, JPN; 3 Cerebrospinal Center, Aoyama General Hospital, Toyokawa, JPN

**Keywords:** cyberknife, intra-articular metastasis, lung cancer, pain relief, palliative treatment, sbrt (stereotactic body radiotherapy), shoulder joint metastasis, shoulder joint pain, shoulder pain, soft tissue metastasis

## Abstract

Intra-articular metastasis of the shoulder (IMS) is extremely rare. Here, we report three cases of IMS from lung cancer treated with stereotactic body radiotherapy (SBRT) using CyberKnife. Case 1 involved a 50-year-old man with IMS of small-cell lung cancer who developed right shoulder pain that worsened over two weeks. The IMS was treated with SBRT in five fractions over eight days (treatment volume: 19.4 mL; prescribed dose: 30 Gy; maximum dose: 51.7 Gy; treatment time: 35 minutes per fraction). Pain relief occurred immediately and resolved completely within 10 days of initiating SBRT. Pain did not recur until the patient died five months later. Case 2 involved a 66-year-old woman with IMS of lung adenocarcinoma who developed left shoulder pain that worsened over one month. The IMS was treated with SBRT in three fractions over three days (treatment volume: 7.5 mL; prescribed dose: 30 Gy; maximum dose: 44.1 Gy; treatment time: 37 minutes per fraction). Pain relief occurred immediately and resolved completely within four days of initiating SBRT. The pain did not recur until the patient died 22 months later. Case 3 involved a 73-year-old man with IMS of lung adenocarcinoma who developed right shoulder pain that worsened over one month. The IMS was treated with SBRT in three fractions over four days (treatment volume: 6.1 mL; prescribed dose: 30 Gy; maximum dose: 46.2 Gy; treatment time: 32 minutes per fraction). Pain relief occurred immediately and resolved completely within one week of initiating SBRT. The pain has not recurred in the nine years and three months since SBRT. No SBRT-related adverse events occurred in any of the cases. IMS should be included in the differential diagnosis of cancer patients with shoulder pain. SBRT may be a useful palliative treatment for such cases.

## Introduction

Shoulder pain is the third most common type of joint pain, following back and knee pain [1]. Common causes of shoulder pain are rotator cuff pathology, adhesive capsulitis, calcific tendinitis, degenerative joint disease, dislocation, fracture, acute trauma, and tumors. Most cases are caused by non-neoplastic conditions, such as frozen shoulder. Although cancer metastasis can occur throughout the body, soft tissue metastasis is significantly rarer than bone metastasis [2,3]. Metastasis to the shoulder joint (SJ) is particularly uncommon; intra-articular metastasis of the shoulder (IMS) is even rarer [4-8]. The treatment strategy for IMS is unclear due to the paucity of reported cases. Stereotactic body radiotherapy (SBRT) is a relatively new radiotherapy technique [9]. The CyberKnife (CK) (Accuray, Sunnyvale, CA, USA) system delivers SBRT with high precision [10]. Here, we report three cases of IMS from lung cancer in which significant pain relief was achieved using CK-based SBRT.

## Case presentation

Case 1

**Figure 1:**
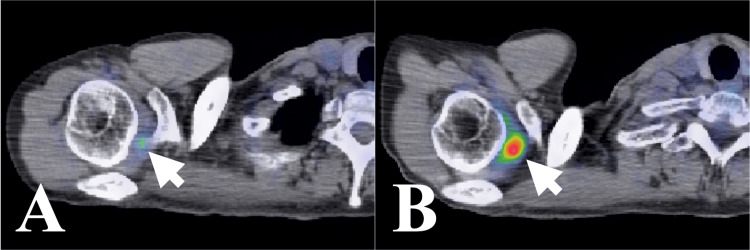
Case 1. Axial FDG-PET/CT findings. (A) Four months before CK-SBRT, a PET/CT scan showing slight focal FDG uptake at the rotator interval of the right shoulder joint. (B) Just before CK-SBRT, a PET/CT scan showing higher focal FDG uptake at the rotator interval of the right shoulder joint. FDG: 2-deoxy-2-[18F] fluoro-D-glucose; PET: positron emission tomography; CT: computed tomography; CK-SBRT: stereotactic body radiotherapy using CyberKnife

**Figure 2:**
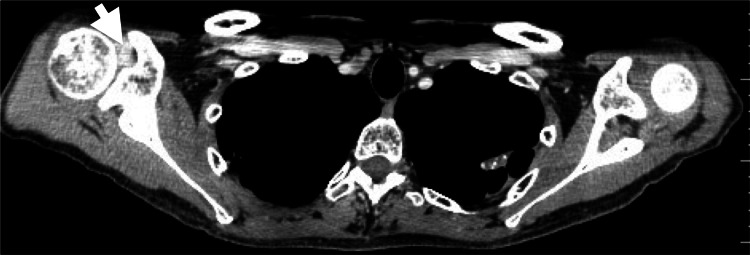
Case 1. Axial CT of CK-SBRT planning showing a contrast-enhanced lesion at the rotator interval of the right shoulder joint. CT: computed tomography; CK-SBRT: stereotactic body radiotherapy using CyberKnife

A 50-year-old man was diagnosed with stage IV small-cell lung cancer two and a half years ago. Two weeks before the presentation, he developed right shoulder pain, which worsened to the point where he had difficulty sleeping at night. Nonsteroidal anti-inflammatory drugs and opioids were ineffective. A positron emission tomography/computed tomography (PET/CT) scan from four months ago showed slight 2-deoxy-2-[18F] fluoro-D-glucose (FDG) uptake in the right SJ (Figure 1A). A repeat PET/CT scan performed following the onset of pain showed increased focal FDG uptake in the right SJ, though no abnormalities were reported by the radiologist (Figure 1B). A subsequent CT scan showed a contrast-enhanced lesion in the right SJ with no abnormalities in the surrounding bones (Figure 2).

**Figure 3:**
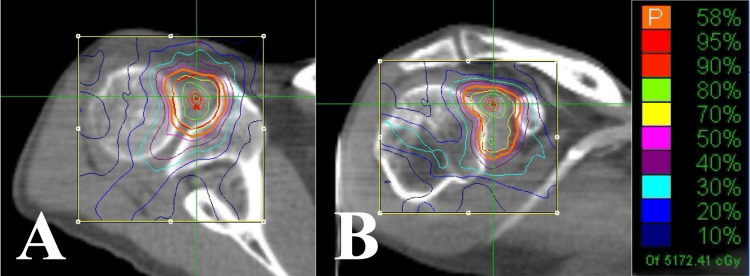
Case 1. Dose distributions of the CK-SBRT plan. (A) Axial section. (B) Coronal section. CK-SBRT: stereotactic body radiotherapy using CyberKnife

Based on the history of cancer, absence of prior shoulder pain, rapid symptom progression, severe drug-resistant pain, and imaging results, the patient was diagnosed with IMS. It was treated with SBRT in five fractions over eight days (treatment volume: 19.4 mL; prescribed dose: 30 Gy; maximum dose: 51.7 Gy; treatment time: 35 minutes per fraction) (Figures 3A, 3B).

Pain relief was immediate after the first session. The following day, the Numerical Rating Scale (NRS) dropped from 10 to 4, and the patient was able to sleep at night. By the end of SBRT, the NRS was 2, and the pain was fully resolved within 10 days of SBRT. The shoulder pain did not recur until the patient died of systemic metastasis five months after SBRT. No SBRT-related adverse events were observed.

Case 2

**Figure 4:**
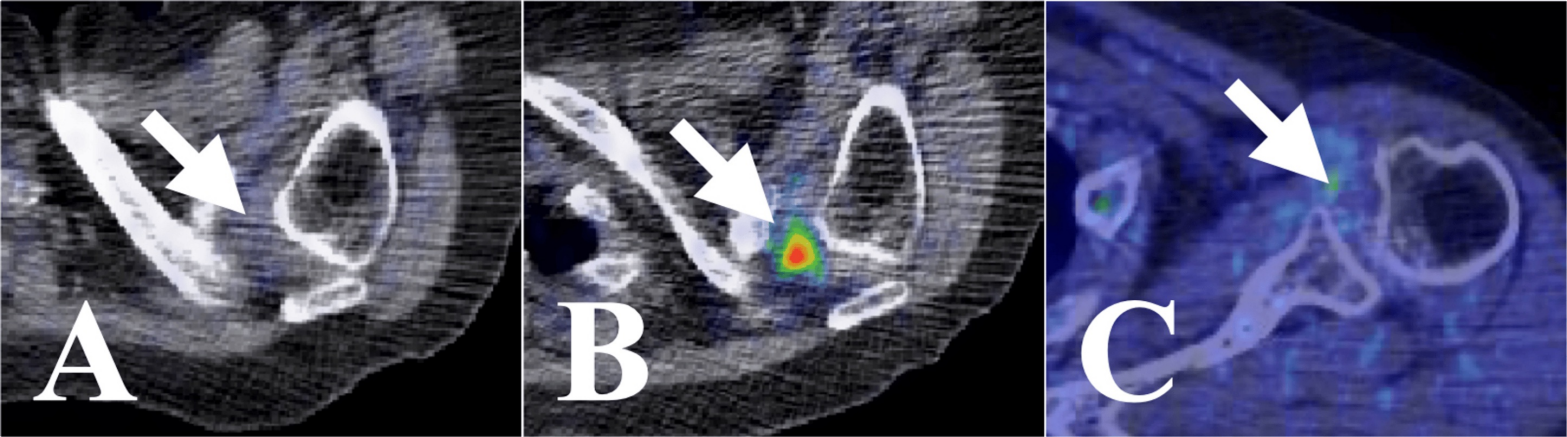
Case 2. Axial FDG-PET/CT findings. (A) Two years and five months before CK-SBRT, a PET/CT scan showing no FDG uptake in the left shoulder joint. (B) Two months before CK-SBRT, a PET/CT scan showing high focal FDG uptake at the rotator interval of the left shoulder joint. (C) Thirteen months after CK-SBRT, a PET/CT scan showing no FDG uptake in the left shoulder joint. FDG: 2-deoxy-2-[18F] fluoro-D-glucose; PET: positron emission tomography; CT: computed tomography; CK-SBRT: stereotactic body radiotherapy using CyberKnife

**Figure 5:**
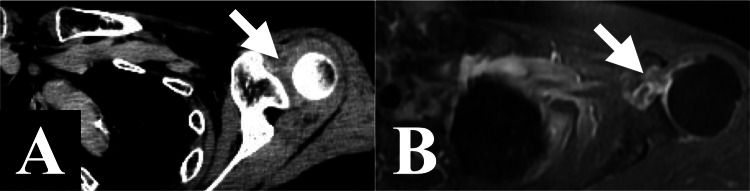
Case 2. Contrast-enhanced CT and MRI findings. (A) Axial CT of CK-SBRT planning showing a contrast-enhanced lesion at the rotator interval of the left shoulder joint. (B) Just before CK-SBRT, axial MRI showing a contrast-enhanced lesion at the rotator interval of the left shoulder joint. CT: computed tomography; MRI: magnetic resonance imaging; CK-SBRT: stereotactic body radiotherapy using CyberKnife

A 66-year-old woman was diagnosed with lung adenocarcinoma (stage IV) with brain metastases two years and eight months ago. One month ago, she developed left shoulder pain when pulling her arms backward, which rapidly worsened. Two years and five months ago, PET/CT revealed no FDG uptake in the left SJ (Figure 4A). However, two months before the onset of pain, PET/CT revealed focal high FDG uptake in the left SJ (Figure 4B). A radiologist diagnosed her with frozen shoulder. Subsequent CT (Figure 5A) and magnetic resonance imaging (MRI) (Figure 5B) showed a contrast-enhanced lesion in the left SJ; however, no abnormalities were observed in the surrounding bones.

**Figure 6:**
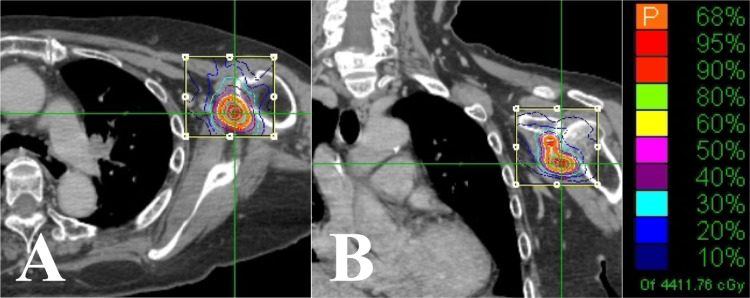
Case 2. Dose distributions of the CK-SBRT plan. (A) Axial section. (B) Coronal section. CK-SBRT: stereotactic body radiotherapy using CyberKnife

Based on the patient’s cancer, absence of prior shoulder pain, rapid progression after onset, severe pain, and imaging findings, we diagnosed her with IMS. The IMS was treated with SBRT in three fractions over three days (treatment volume: 7.5 mL; prescribed dose: 30 Gy; maximum dose: 44.1 Gy; treatment time: 37 minutes per fraction) (Figure 6).

The pain was relieved immediately after the initial SBRT session. The NRS decreased from 10 to 4, and the pain was reduced when she pulled her upper arm backward. At the end of SBRT, the NRS decreased to 2, and the shoulder pain disappeared the following day (day four). Thirteen months after SBRT, PET/CT revealed no FDG uptake in the left SJ (Figure 4C). The shoulder pain did not recur until 22 months following SBRT, when the primary cancer progressed and the patient died. No SBRT-related adverse events occurred.

Case 3

**Figure 7:**
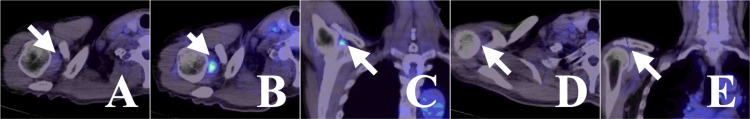
Case 3. FDG-PET/CT findings. (A) Eight months before CK-SBRT, a PET/CT scan showing no FDG uptake in the right shoulder joint. (B) Axial and (C) coronal section, just before CK-SBRT, a PET/CT scan showing high focal FDG uptake at the rotator interval of the right shoulder joint. (D) Axial and (E) coronal section, six months after CK-SBRT, a PET/CT scan showing no FDG uptake in the right shoulder joint. FDG: 2-deoxy-2-[18F] fluoro-D-glucose; PET: positron emission tomography; CT: computed tomography; CK-SBRT: stereotactic body radiotherapy using CyberKnife

**Figure 8:**
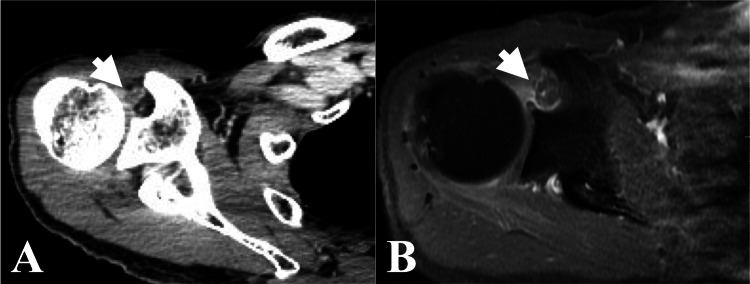
Case 3. Contrast-enhanced CT and MRI findings. (A) Axial CT of CK-SBRT planning showing a contrast-enhanced lesion at the rotator interval of the right shoulder joint. (B) Just before CK-SBRT, axial MRI showing a contrast-enhanced lesion at the rotator interval of the right shoulder joint. CT: computed tomography; MRI: magnetic resonance imaging; CK-SBRT: stereotactic body radiotherapy using CyberKnife

A 73-year-old man was diagnosed with lung adenocarcinoma (stage IV) with brain metastases 1 year and 11 months ago. One month ago, he began to feel pain in his right shoulder when dressing, and the pain worsened. Eight months ago, PET/CT showed no FDG uptake in the right SJ (Figure 7A). PET/CT after the onset of right shoulder pain showed focal high FDG uptake in the right SJ; however, the radiologist diagnosed it as scapular metastasis (Figures 7B, 7C). CT (Figure 8A) and MRI (Figure 8B) showed a contrast-enhanced lesion in the right SJ, but no abnormalities were found in the surrounding bones.

**Figure 9:**
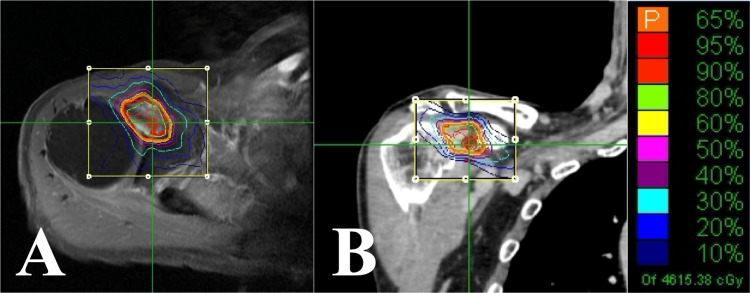
Case 3. Dose distribution of the CK-SBRT plan. (A) Axial section. (B) Coronal section. CK-SBRT: stereotactic body radiotherapy using CyberKnife

Based on the patient’s cancer history, absence of prior shoulder pain, rapid progression from onset, severe pain, and imaging findings, we diagnosed the patient with IMS. The IMS was treated with SBRT in three fractions over four days (treatment volume: 6.1 mL; prescribed dose: 30 Gy; maximum dose: 46.2 Gy; treatment time: 32 minutes per fraction) (Figure 9).

The pain was relieved immediately after the initial SBRT session. At the end of SBRT, the NRS decreased from 10 to 4. One week after the start of SBRT, the shoulder pain disappeared. Six months after SBRT, PET/CT showed no FDG uptake in the right SJ (Figures 7D, 7E). Up to eight years and four months following SBRT, PET/CT was performed six times; however, no FDG uptake was observed in the right SJ. At the time of the final examination, nine years and three months after SBRT, the right shoulder pain had not recurred. No SBRT-related adverse events occurred.

## Discussion

Joints metastases are rare, with most reported cases occurring in the knee joint [7,11]. SJ metastasis is rare, and IMS without adjacent bone metastasis is even rarer [12]. Most intra-articular metastases occur in the synovial tissue. To our knowledge, only four cases of synovial metastasis in SJ have been reported [5-8]. One of these four cases may not represent a true synovial metastasis due to concurrent involvement of the humerus [8]. Diagnosing shoulder metastasis in patients presenting with shoulder pain can be challenging in the absence of tissue diagnosis. Sano et al. reported that 9 of 34 patients (26%) with malignant shoulder tumors were initially misdiagnosed as having frozen shoulder [13]. In our three cases, IMS was not identified by the radiologist on FDG-PET/CT. The diagnosis of IMS was based on the following criteria: (1) a known history of malignancy, (2) no history of prior shoulder pain, such as frozen shoulder, (3) rapid worsening of pain after onset, (4) focal high FDG uptake in the SJ on PET, (5) contrast-enhanced CT and MRI showed tumor lesions in the SJ, and (6) no abnormalities in the surrounding bones. These were common features in all three cases. Furthermore, the following information would be useful for the diagnosis: in Case 1, analgesics provided no pain relief, and in Cases 1 and 3, there was no restriction on shoulder movement. Limited shoulder movement is one of the main symptoms of frozen shoulder, the most common type of shoulder pain.

Fluid cytology or tissue biopsy is an important examination for a definitive diagnosis. A limitation of the present study is the lack of a histopathological confirmation. Patients with synovial metastasis generally have a poor prognosis, with a median survival time of fewer than five months [5,12,14]. The treatment goal is palliative care, and our patients did not wish to undergo invasive examinations. Therefore, in our cases, we did not perform these examinations. In Cases 2 and 3, we considered these to be metastatic lesions because the focal high FDG uptake disappeared on PET following SBRT. It is important to include neoplastic diseases in the differential diagnosis of shoulder pain. Even in non-neoplastic diseases of the shoulder joint, FDG uptake on PET and contrast enhancement on MRI have been reported; caution is required in imaging diagnosis [15-18]. A comprehensive judgment is required based on medical history, clinical symptoms, FDG-PET, CT, and MRI. Currently, there is no established treatment protocol for IMS. Surgical resection of IMS is invasive; therefore, chemotherapy and radiotherapy are usually the primary treatment options. Recently, advances in cancer treatment have increased long-term survival even in stage IV patients. We have one patient in the clinic who survived for an extremely long time while maintaining a good quality of life (QOL). Even for patients with stage IV cancer, a treatment plan that considers long-term QOL is necessary.

Radiotherapy is a minimally invasive treatment that can be performed on elderly patients and those in poor general condition. However, in radiotherapy, adverse events may occur even with low doses of radiation. Khurram et al. reported that a patient received palliative external beam radiotherapy (a total of two sessions of 8 Gy per fraction) to his synovial metastasis of the knee for pain relief; this resulted in him having significant stiffness and mobility issues due to radiation fibrosis [19]. SBRT is a cancer treatment modality that delivers highly precise and powerful radiation to cancer cells while minimizing damage to the surrounding normal tissue [9]. CK is an image-guided SBRT device that consists of a robotic arm, a linear accelerator, and a target tracking system [10]. The CK system uses a wide range of motion to move the robotic arm to irradiate the target while minimizing damage to nearby organs [10]. SBRT is a minimally invasive treatment compared to standard surgery, has a shorter treatment period than conventional radiotherapy, and is proven to be more effective. The advantages of SBRT for IMS are a short treatment period, early treatment effect, and reduced adverse effects on joint function due to inflammation and adhesion. In our cases, shoulder pain was relieved immediately after the initial SBRT treatment, demonstrating an extremely rapid relief. Our Case 3 had maintained the pain-relieving effect for nearly 10 years without an SBRT-related adverse event. Thus, our reported cases suggest that SBRT might be a useful palliative treatment option for IMS. Problems with SBRT include the long treatment time and the technical skill required to create a trunk fixation device and reproduce the position at the beginning of treatment. In our cases, all patients were immobilized in a supine position using Vac-Lok (CIVCO, USA). The indications, optimal dose, and fraction number, and other details of SBRT for IMS have not been established. It is essential to accumulate detailed clinical information to determine treatment strategies because of the limited number of reported IMS cases.

## Conclusions

We described three cases in which palliative SBRT was administered to treat IMS due to lung cancer. IMS may be overlooked; it should be considered in the differential diagnosis of shoulder pain. A comprehensive evaluation of shoulder pain necessitates a multifaceted approach that incorporates medical history, clinical presentation, FDG-PET, CT, MRI, and other relevant diagnostic modalities. SBRT is a promising treatment option due to its potential for early pain relief and favorable safety profile in patients with IMS. However, the specifics of SBRT for IMS, including differential diagnosis, treatment indications, optimal dose, number of fractions, and treatment duration, have yet to be established. It is essential to gather additional clinical data to evaluate the efficacy of SBRT as a treatment option for IMS.

## References

[1] Badley EM, Tennant A (1992). Changing profile of joint disorders with age: findings from a postal survey of the population of Calderdale, West Yorkshire, United Kingdom. Ann Rheum Dis.

[2] Watmough PJ, Canty SJ, Higgins G (2005). Soft tissues metastases from malignant tumors. Orthop Proceed.

[3] Plaza JA, Perez-Montiel D, Mayerson J, Morrison C, Suster S (2008). Metastases to soft tissue: a review of 118 cases over a 30-year period. Cancer.

[4] Kawahara K, Kitagawa T, Takagi K (1983). A clinical study of shoulder girdle tumors. Orthop Traumatol.

[5] Benhamou CL, Tourliere D, Brigant S, Maitre F, Cauderlier P (1988). Synovial metastasis of an adenocarcinoma presenting as a shoulder monoarthritis. J Rheumatol.

[6] Morbidi M, Magnani M, Della Rocca C (1998). Synovial metastasis of the shoulder detected by arthroscopy as the presenting manifestation of lung adenocarcinoma. Arthroscopy.

[7] Capovilla M, Durlach A, Fourati E (2007). Chronic monoarthritis and previous history of cancer: think about synovial metastasis. Clin Rheumatol.

[8] Aloui I, Njim L, Moussa A, Hamdi MF, Abid A, Zakhama A (2009). Shoulder arthritis as a lung metastatic carcinoma revealer. A case report. Orthop Traumatol Surg Res.

[9] Ma L, Wang L, Tseng CL, Sahgal A (2017). Emerging technologies in stereotactic body radiotherapy. Chin Clin Oncol.

[10] Adler JR Jr, Chang SD, Murphy MJ, Doty J, Geis P, Hancock SL (1997). The Cyberknife: a frameless robotic system for radiosurgery. Stereotact Funct Neurosurg.

[11] Younes M, Hayem G, Brissaud P, Grossin M, Kahn MF, Meyer O (2002). Monoarthritis secondary to joint metastasis. Two case reports and literature review. Joint Bone Spine.

[12] Thompson KS, Reyes CV, Jensen J, Gattuso P, Sacks R (1996). Synovial metastasis: diagnosis by fine-needle aspiration cytologic investigation. Diagn Cytopathol.

[13] Sano H, Hatori M, Mineta M, Hosaka M, Itoi E (2010). Tumors masked as frozen shoulders: a retrospective analysis. J Shoulder Elbow Surg.

[14] Levine HR, Tingle E, Carter B, Dockery D (2013). Synovial metastasis from lung cancer. Proc (Bayl Univ Med Cent).

[15] Moon YL, Lee SH, Park SY, Yu JC, Gorthi V (2010). Evaluation of shoulder disorders by 2-[F-18]-fluoro-2-deoxy-D-glucose positron emission tomography and computed tomography. Clin Orthop Surg.

[16] Salem U, Zhang L, Jorgensen JL, Kumar R, Amini B (2015). Adhesive capsulitis mimicking metastasis on 18F-FDG-PET/CT. Clin Nucl Med.

[17] Sridharan R, Engle MP, Garg N, Wei W, Amini B (2017). Focal uptake at the rotator interval or inferior capsule of shoulder on (18)F-FDG PET/CT is associated with adhesive capsulitis. Skeletal Radiol.

[18] Tamai K, Hamada J, Nagase Y, Morishige M, Naito M, Asai H, Tanaka S (2024). Can magnetic resonance imaging distinguish clinical stages of frozen shoulder? A state-of-the-art review. JSES Rev Rep Tech.

[19] Khurram R, Khurram A, Chaudhary K (2020). Index case of synovial metastasis in a patient with transitional cell carcinoma of the bladder. BMJ Case Rep.

